# Partial Heart Transplantation Promotes Organ Stewardship: Domino Hearts and Split Roots

**DOI:** 10.1016/j.atssr.2024.07.033

**Published:** 2024-08-30

**Authors:** Berk Aykut, Douglas M. Overbey, Cathlyn K. Medina, Tariq M. Omer, T. Konrad Rajab, Smith M. Ngeve, Ziv Beckerman, Joseph W. Turek

**Affiliations:** 1 Duke Congenital Heart Surgery Research and Training Laboratory, Durham, North Carolina; 2 Department of Surgery, Duke University Hospital, Durham, North Carolina; 3 Duke University School of Medicine, Duke University, Durham, North Carolina; 4 Arkansas Children’s Hospital, Little Rock, Arkansas

## Abstract

**Background:**

Partial heart transplantation (PHT) has emerged as a pioneering approach for treating infants with irreparable heart valve dysfunction. However, the scarcity of suitable donors presents a significant bottleneck to its widespread application. This study introduces and evaluates the novel use of domino and split-root procedures within PHT.

**Methods:**

We describe 6 pediatric cardiac patients who underwent either domino or split-root PHT at our institution.

**Results:**

From May to August 2023, our team successfully executed 3 domino and 3 split-root PHTs, including 1 procedure that involved interinstitutional collaboration. These cases highlight the procedural feasibility and the potential for broader application.

**Conclusions:**

The implementation of PHT represents a significant advance in pediatric heart care. Domino and split-root techniques within the PHT framework have the potential to substantially increase both donor availability and recipient capacity. These strategies usher in a new era of organ stewardship through addressing the challenge of donor organ shortage.

**In Short**

▪ Domino and split-root procedures build on the transformative approach of partial heart transplantation to pediatric cardiac care.
▪ Domino and split-root procedures embody a new era of medical innovation and organ stewardship.

The management of heart valve dysfunction in neonates poses a significant medical challenge because of the absence of growth-accommodating heart valve implants. Standard treatments, such as cadaveric homografts, are constrained by their nonviable nature, which precludes growth and self-repair, necessitating numerous surgical interventions until a permanent, adult-sized valve can be accommodated. This iterative approach is fraught with complications, leading to suboptimal clinical outcomes. In response to this dilemma, partial heart transplantation (PHT) has emerged as an innovative therapeutic strategy.^1^ PHT offers a targeted solution by transplanting only the portion of the heart containing the outflow valves, thereby preserving the native ventricles and enabling continued growth in developing children. Our group recently documented the inaugural application of PHT using a neonatal donor’s aortic and pulmonary valves to address truncus arteriosus and an accompanying dysfunctional truncal valve.^2^ This novel intervention not only serves as a technical advancement but also embodies a profound ethic of organ stewardship. On one hand, it empowers bereaved families to transform their personal loss into a legacy of life, offering other children a chance for improved health. On the other, it redefines recipient care by judiciously using donor hearts that would be deemed unsuitable for full transplantation, thereby extending the utility of available organs. Despite its promise, PHT is not immune to the pervasive challenge of donor shortage that plagues the field of organ transplantation. The deficit of available donors for PHT necessitates the exploration and implementation of organ utilization strategies. Among such strategies is the domino procedure, a surgical innovation traditionally employed in multiple-organ transplants that involves harvesting viable organs from transplant recipients for use in another recipient.^3^ This method has seen success across various transplant scenarios, including heart-kidney, heart-lung, and heart-liver transplants, and is revered for its potential to significantly amplify the donor organ pool. The seeds of domino use in PHT were planted in the seminal work by Yacoub and others for freshly procured donor valves placed in the aortic position.^4^^,^^5^ These recipients were not, however, immunosuppressed, as in the PHT technique. In parallel, the split-root transplant approach capitalizes on the functional segments of a single heart to benefit multiple recipients, effectively doubling the reach of a single donor heart. By allowing lifesaving procedures to be extended to a greater number of children, these methods exemplify a new paradigm in organ stewardship and transplantation. In this article, we describe the application of the domino and split-root techniques within the context of PHT. These pioneering strategies not only highlight the evolution of a new surgical paradigm but also underscore a commitment to maximizing the impact of organ donation. By enhancing the distribution of viable valve implants, these techniques could significantly broaden the therapeutic prospects for neonates in dire need of heart valves or other heart tissue, ultimately advancing the frontier of care for children with congenital heart disease.

## Methods

### Domino Procedure

**Figure 1:**
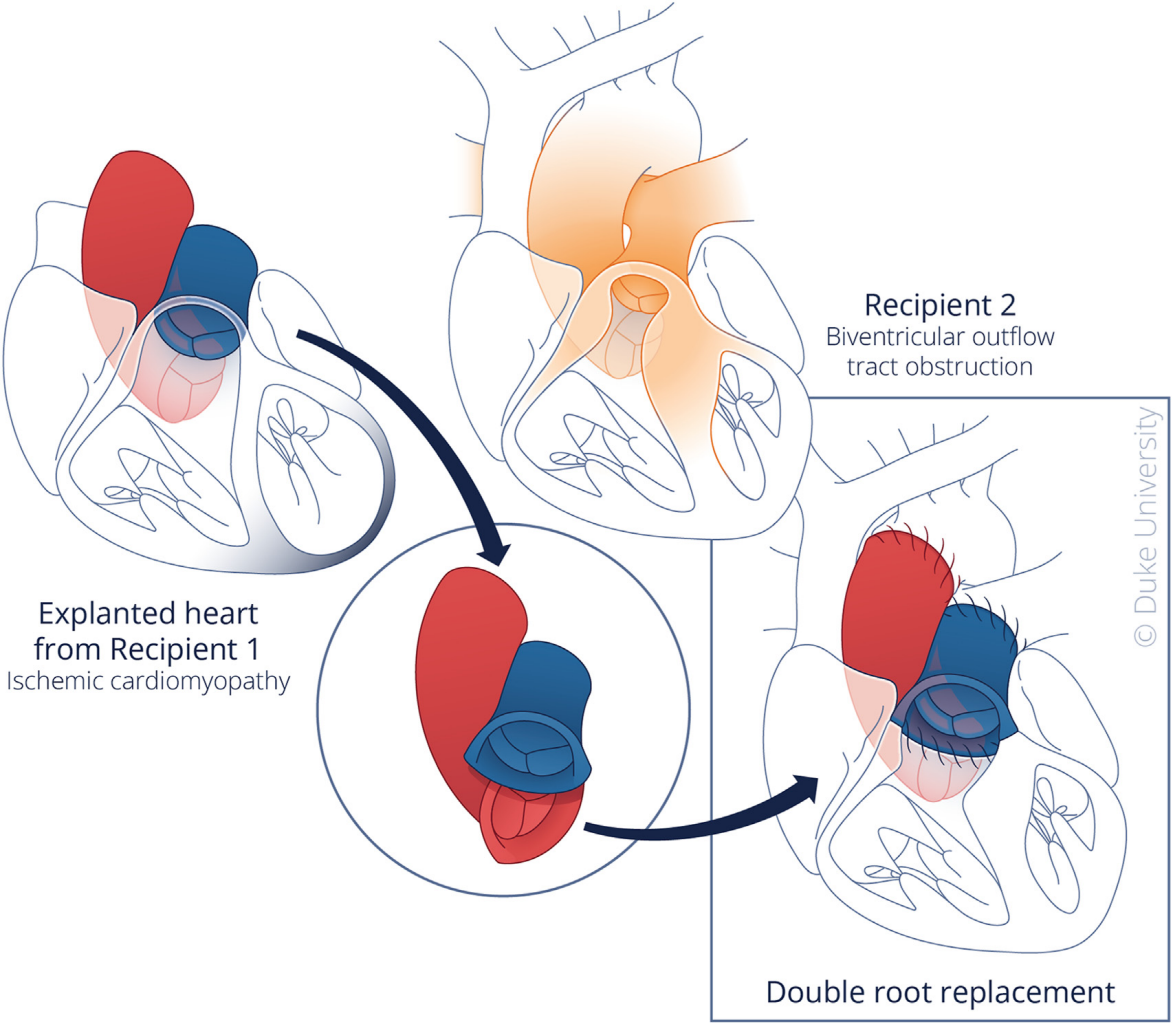
Conceptual schematic depicting domino partial heart transplantation. Illustrated by Megan Llewellyn, MSMI; copyright Duke University; with permission under a CC BY-ND 4.0 license.

In the domino PHT procedure, we identified orthotopic heart transplant recipients with healthy semilunar valves. After explantation of the native heart, the aortic and pulmonary roots were procured for transplantation into PHT recipients (Figure 1).

The first donor was a 4-day-old, 3.5-kg neonate who underwent donation after cardiac death (DCD). The heart from this neonate was transplanted into a 3-month-old, 3.6-kg infant suffering from ischemic cardiomyopathy, yet with retained functional semilunar valves. The explanted heart of this infant was designated suitable for subsequent root donation, and informed consent for this donation was obtained after discussion with the parents. During the explantation, additional lengths of both the pulmonary artery and aorta were procured, with the valvular roots subsequently preserved in cold storage using cardioplegia and ice slush. The next day, a second recipient, a 3-month-old, 3.8-kg infant with biventricular outflow tract obstruction, underwent a successful double root transplant, using the semilunar valves procured from the initial recipient.

The second case involved a pulmonary root transplant in a 4-month-old, 5.0-kg female patient diagnosed with tetralogy of Fallot and pulmonary atresia. The patient had previously undergone placement of a ductal stent. The donor of the living root was a 1-month-old infant weighing 4.3 kg, diagnosed with hypoplastic left heart syndrome, with a functioning pulmonary valve. This infant received a DCD heart from an 8-day-old neonate who weighed 3.9 kg.

In the third case, a 3-month-old, 5.3-kg male patient, also presenting with tetralogy of Fallot and pulmonary atresia, had undergone ductal stent placement. The donor of the pulmonary root was a 6-month-old infant weighing 8 kg, diagnosed with left coronary atresia, with functioning semilunar valves. This infant received a DCD heart from a 5-month-old neonate who weighed 7 kg. Table 1 highlights the characteristics for domino PHT donors and recipients.

**Table 1:** Patient Characteristics for Domino Transplantation

Domino Procedure	Cardiac Allograft Donor	Living Root Donor	Recipient	Outcome
Domino 1	3.5-kg, 4-day-old DCD donor with healthy semilunar valves	3.6-kg, 3-month-old with ischemic cardiomyopathy with functional semilunar valves	3.8-kg, 3-month-old boy with biventricular outflow tract obstruction	Double root replacement (6.3-mm aortic root and 8-mm pulmonary root); uncomplicated postoperative course with discharge to home on POD 36
Domino 2	3.9-kg, 8-day-old DCD donor with healthy semilunar valves	4.3-kg, 1-month-old with hypoplastic left heart syndrome with functional pulmonary valve	5.0-kg, 4-month-old girl with tetralogy of Fallot with membranous pulmonary atresia	Pulmonary root replacement (10 mm); uncomplicated postoperative course with discharge to home on POD 13
Domino 3	7.0-kg, 5-month-old DCD donor with healthy semilunar valves	8-kg, 6-month-old with left coronary atresia with functional semilunar valves	5.3-kg, 3-month-old boy with tetralogy of Fallot with membranous pulmonary atresia	Pulmonary root replacement (9.6 mm); uncomplicated postoperative course with discharge to home on POD 9

DCD, donation after cardiac death; POD, postoperative day.

### Split-Root Procedure

**Figure 2:**
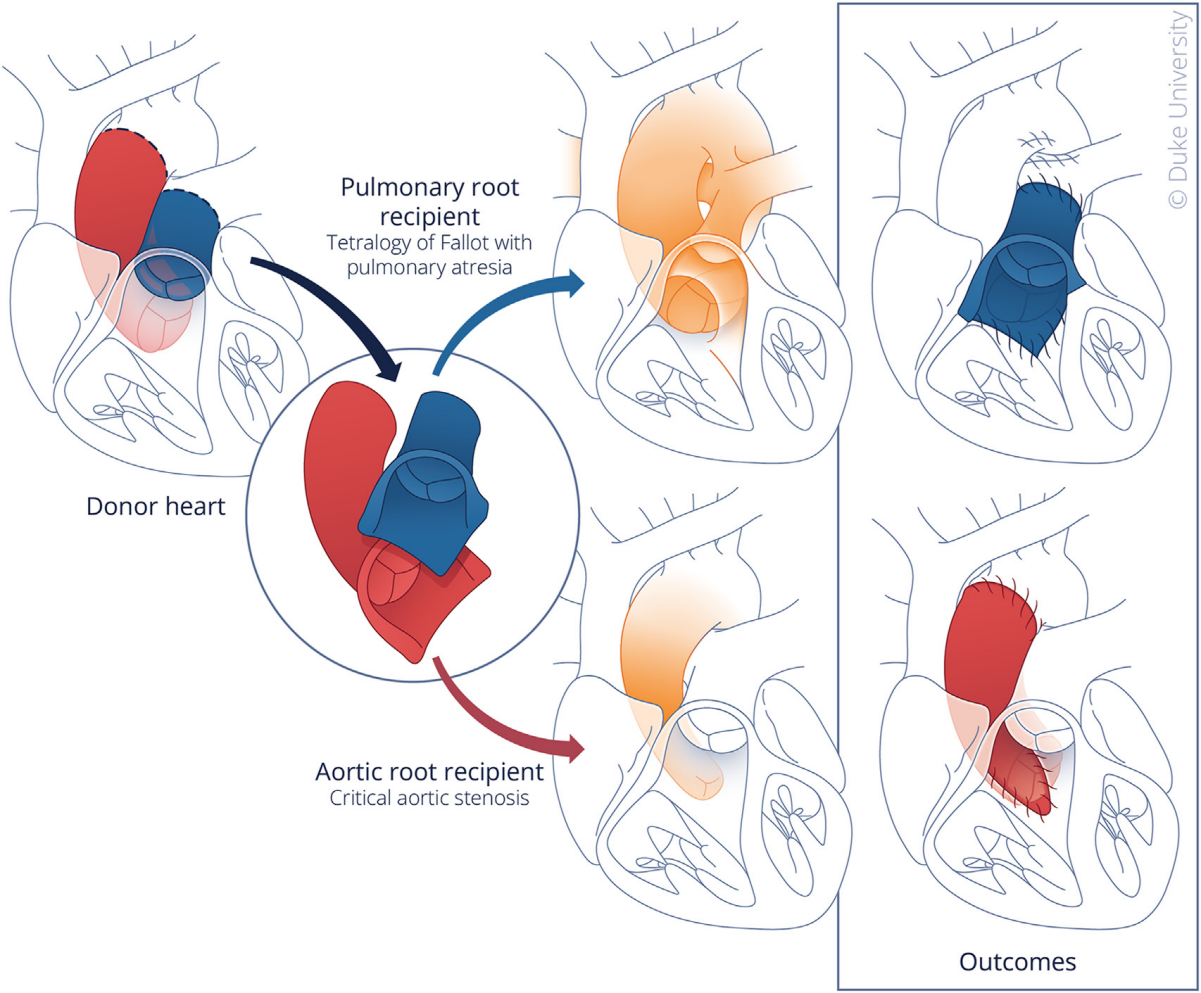
Conceptual schematic depicting split-root partial heart transplantation. Illustrated by Megan Llewellyn, MSMI; copyright Duke University; with permission under a CC BY-ND 4.0 license.

To use the split-root procedure, we maximize a single donor heart, initially deemed nonviable for conventional transplantation, to perform PHT in 2 infants (Figure 2). Our first split-root case featured a 6-day-old neonate, weighing 3.2 kg, with a diagnosis of truncus arteriosus and an interrupted arch with moderate truncal valve insufficiency. The donor heart, harvested 1 day earlier by another institution using the pulmonary root, had the aortic root preserved under cold storage and transported to Duke. Following preparation, which included ligation of the coronary arteries and repair of a vessel injury, the truncal valve was successfully repaired. This allowed the integration of the living aortic root into the right ventricle to pulmonary artery position. The donor was a 9-month-old infant with a weight of 3.4 kg who had succumbed to hypoxic brain injury.

The donor for our second and third cases was a 12-day-old neonate with a weight of 3.4 kg who had succumbed to cardiac death. The organ procurement was coordinated directly with the respective organizations, ensuring the integrity of the aortic and pulmonic homografts. The organ was preserved in cold storage to maintain its viability before transplantation. The second split-root procedure involved a 12-day-old neonate, matching the donor's weight, diagnosed prenatally with critical congenital aortic stenosis unamenable to balloon dilation. The corrective surgical procedure entailed living aortic root replacement and Konno root enlargement procedure. The pulmonary root was implanted in a 2-month-old infant, weighing 2.8 kg, with a diagnosis of tetralogy of Fallot, pulmonary atresia, and ventricular septal defect, representing our third split-root procedure. Following preparation, which included closure of the malignment ventricular septal defect and right ventricular outflow tract resection, the living pulmonary conduit was placed into the right ventricle to pulmonary artery position. Table 2 highlights the characteristics for split-root PHT donors and recipients.

**Table 2:** Patient Characteristics for Split-Root Transplantation

Split-Root Procedure	Donor	Recipient	Outcomes
Interinstitutional split-root recipient 1	Procured by another institution	3.2-kg, 6-day-old male neonate with truncus arteriosus and interrupted arch	RV-PA conduit with living aortic homograft (7.5 mm); uncomplicated postoperative course with discharge to home on POD 16
Split-root recipient 2	3.4-kg, 12-day-old DCD donor	3.3-kg, 12-day-old male neonate with critical aortic stenosis	Living aortic root replacement with aortic homograft (11 mm); postoperative course complicated by hemodynamic instability requiring vasopressors and stress-dose corticosteroids; discharge to home on POD 41
Split-root recipient 3	3.4-kg, 12-day-old DCD donor	2.8-kg, 2-month-old female infant with tetralogy of Fallot, pulmonary atresia, and VSD	RV-PA conduit with living pulmonary homograft (11 mm); uncomplicated postoperative course with discharge to home on POD 21

DCD, donation after cardiac death; PA, pulmonary artery; POD, postoperative day; RV, right ventricle; VSD, ventricular septal defect.

## Results

Between May and August 2023, our team performed 3 domino and 3 split-root PHTs, including one conducted in collaboration with another institution. These cases demonstrate both the feasibility of the procedure and its potential for wider application.

## Comment

Current valve implantation technologies are limited by their inability to grow with the child. This limitation often results in multiple surgical interventions during the course of a child’s development, causing significant morbidity and mortality.^6^ PHT addresses this limitation by using living heart tissues capable of growth, harnessing the body’s innate regenerative capabilities to minimize the need for subsequent operations.^1^^,^^2^ The potential for PHT to offer durable, growing valve replacements is a beacon of hope for pediatric patients suffering from valvular dysfunction that is not amenable to surgical repair. The advent of the domino and split-root transplant procedures signals the natural evolution in this new field of PHT, offering viable solutions to the critical shortage of suitable donor hearts. These procedures, by virtue of their design, have the potential not only to amplify the availability of donor organs but also to improve the long-term outcomes of pediatric heart valve implants. The domino PHT procedure provides an intriguing method to mitigate organ scarcity.^7^ By harvesting viable valves from the hearts of orthotopic transplant recipients, this technique offers a regenerative approach that could see the number of pediatric patients benefiting from a single donor heart multiply. Use of approximately 500 pediatric orthotopic heart transplant cardiectomies in the United States could stand to have an impact on hundreds of pediatric heart patients annually. This approach is further augmented by the potential use of valves from hearts unsuitable for full orthotopic transplantation because of poor ventricular function, thus reinforcing the cycle of life through organ stewardship.^8^^,^^9^ The establishment of a centralized allocation system will be of paramount importance in this context, ensuring that the finite supply of donor organs is distributed equitably and effectively, thereby optimizing the impact of every PHT performed. Complementing the domino technique, the split-root procedure directly addresses the exigent need for cardiac tissues by bifurcating a single donor heart into multiple viable components. This division, far from being a mere act of surgical procedure, represents a paradigm shift in organ utilization, effectively expanding the donor pool and providing a lifeline to numerous infants in need of growing valves.^10^ Whereas currently this has only been performed clinically using the semilunar valves, early laboratory investigations suggest atrioventricular valves and cardiac patch tissue could play a role in the future of PHT. Thus, the evolution of this field could conceivably see a day in which 1 donor heart aids 3 or more recipients.

In summary, the domino and split-root procedures build on the transformative approach of PHT to pediatric cardiac care. By prospectively multiplying the number of pediatric patients who can benefit from lifesaving heart tissue implants, these strategies embody a new era of medical innovation and organ stewardship. Although ongoing research, refinement, and broader implementation of these procedures will be critical, the future of PHT, through these advances, is set to offer a beacon of hope for pediatric heart patients and their families worldwide.

## References

[1] Rajab T.K., Vogel A.D., Turek J.W. (2024). Partial heart transplantation: a new option for paediatric heart valve replacement. Nat Rev Cardiol.

[2] Turek J.W., Kang L., Overbey D.M., Carboni M.P., Rajab T.K. (2024). Partial heart transplant in a neonate with irreparable truncal valve dysfunction. JAMA.

[3] Maynes E.J., O'Malley T.J., Austin M.A. (2020). Domino heart transplant following heart-lung transplantation: a systematic review and meta-analysis. Ann Cardiothorac Surg.

[4] Mehrotra R., Srivastava S., Airan B. (1997). Aortic valve replacement with a homovital valve. Tex Heart Inst J.

[5] Yacoub M., Rasmi N.R., Sundt T.M. (1995). Fourteen-year experience with homovital homografts for aortic valve replacement. J Thorac Cardiovasc Surg.

[6] Henaine R., Roubertie F., Vergnat M., Ninet J. (2012). Valve replacement in children: a challenge for a whole life. Arch Cardiovasc Dis.

[7] Cochrane A.D., Smith J.A., Esmore D.S. (1991). The "domino-donor" operation in heart and lung transplantation. Med J Aust.

[8] Quintao R., Kwon J.H., Bishara K., Rajab T.K. (2023). Donor supply for partial heart transplantation in the United States. Clin Transplant.

[9] Shugh S.B., Szugye N.A., Zafar F. (2020). Expanding the donor pool for congenital heart disease transplant candidates by implementing 3D imaging-derived total cardiac volumes. Pediatr Transplant.

[10] Stewart D., Hasz R., Lonze B. (2023). Beyond donation to organ utilization in the USA. Curr Opin Organ Transplant.

